# Stability Analysis for a Multi-Camera Photogrammetric System

**DOI:** 10.3390/s140815084

**Published:** 2014-08-18

**Authors:** Ayman Habib, Ivan Detchev, Eunju Kwak

**Affiliations:** 1 Department of Geomatics Engineering, University of Calgary, 2500 University Drive NW, Calgary, AB, Canada T2N 1N4; E-Mail: ahabib@ucalgary.ca; 2 NovAtel Inc., 1120 68th Avenue NE, Calgary, AB, Canada T2E 8S5; E-Mail: eunju.kwak@novatel.com

**Keywords:** system calibration, simulation-based stability analysis, forward/backward projection, image/object space parallax, normalized coordinate generation

## Abstract

Consumer-grade digital cameras suffer from geometrical instability that may cause problems when used in photogrammetric applications. This paper provides a comprehensive review of this issue of interior orientation parameter variation over time, it explains the common ways used for coping with the issue, and describes the existing methods for performing stability analysis for a single camera. The paper then points out the lack of coverage of stability analysis for multi-camera systems, suggests a modification of the collinearity model to be used for the calibration of an entire photogrammetric system, and proposes three methods for system stability analysis. The proposed methods explore the impact of the changes in interior orientation and relative orientation/mounting parameters on the reconstruction process. Rather than relying on ground truth in real datasets to check the system calibration stability, the proposed methods are simulation-based. Experiment results are shown, where a multi-camera photogrammetric system was calibrated three times, and stability analysis was performed on the system calibration parameters from the three sessions. The proposed simulation-based methods provided results that were compatible with a real-data based approach for evaluating the impact of changes in the system calibration parameters on the three-dimensional reconstruction.

## 1. Introduction

The low cost and the off-the-shelf availability of consumer-grade digital cameras have caused their wide-spread use in photogrammetric applications. The end goal of most photogrammetric applications is the generation of accurate 3D point coordinates for an object of interest or a mapped scene. One of the factors for achieving high quality reconstruction is the knowledge of the interior orientation parameters (IOPs). The IOPs are derived from a geometric camera calibration. For metric film cameras, this is accomplished by the manufacturer or a special calibration agency with the use of multi-collimators. For consumer-grade digital cameras, the preferred procedure is via a bundle adjustment with self-calibration [1–3], the foundations of which were given by Brown [4,5]. The former approach takes place in a laboratory setting, while the latter one could be either in a laboratory or *in-situ*, depending on the project circumstances. The IOPs describe the location of the perspective centre relative to the image plane together with various distortions along the image plane. Connecting the perspective centre with distortion-free points in image space defines a bundle of light rays. In effect, the target function of camera calibration is to achieve similarity of the reconstructed bundle to the incident one at the moment of exposure. Examples for the calibration of low-cost off-the-shelf digital cameras can be found in Chandler *et al.* [6] and Fraser [7]. The next two subsections will address the concepts of stability analysis of a single camera and stability analysis of a multi-camera system.

### 1.1. Stability Analysis of a Single Camera

**Figure 1.**
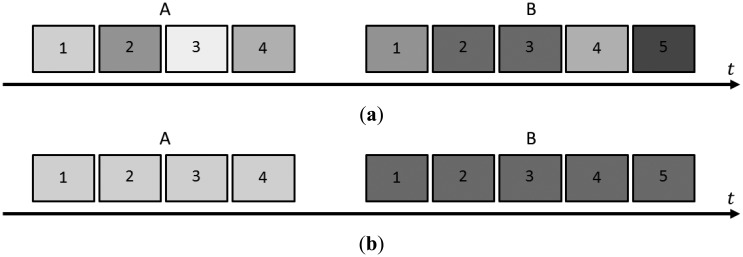
Examples of photo variant (**a**) and block invariant (**b**) variations in the IOPs; the different shades of grey represent IOP sets with significant changes.

Simply calibrating a camera once or “every once in a while” may not be enough to achieve the desired object space reconstruction accuracy. Since consumer-grade digital cameras are not designed with photogrammetric applications in mind, their internal geometry may vary over time. Variations in the IOPs can be *intentional* or *unintended*. Intentional variations are usually due to the mode of operation, e.g., focusing every photo. Unintended variations are caused by the structural instability of the camera. In both cases, mechanical movements (e.g., sensor within the camera body, within the lens “tube” or the lens mount), routine handling, transportation, disassembly and reassembly of the lens from the camera body or other external forces, thermal effects, and influences in the processing chain play a role in changing the internal geometry [8]. Small, and hopefully insignificant, variations in the IOPs may occur within the course of a single data collection campaign. These variations are referred to as photo variant (the term used in the cited literature is actually “photo invariant”; however, the authors consider the term “photo variant” to be more appropriate; see Figure 1a) [9]. Technically, different IOP parameters must be used for each photo, but in practice this is often ignored. If the variations of the IOPs in each photo are not ignored but are solved for, the solution will not be reliable due to the correlations among the many parameters involved in the estimation process. Larger, and most likely significant, variations in the IOPs may occur between data collection campaigns. Those variations are referred to as block invariant (see Figure 1b), and the same IOP parameters must be applied to all photos within a certain block (*i.e.*, a set of photos) [9].

There are two approaches for coping with the geometric instability of a camera. One of them is through parameterization, and the other one through mechanical stabilization [8]. The first one allows for photo variant orientation or introduces an extended set of additional parameters to the standard set of IOPs. For example, Hastedt *et al.* [10] used different principal distance and principal point offset for each collected image with a correction model based on finite element analysis; and Läbe and Förstner [11] added parameters for the range of the principal distance, the changes in the principal point coordinates, and distortions over the image format. Extended sets of additional parameters to model geometrical instability for camera calibration should, however, only be used for strong networks with lots of redundancy, *i.e.*, in high precision applications. Instead of introducing approaches to cope with temporal changes in the IOPs within the same data acquisition exercise, it is recommended to perform mechanical stabilization, and use the standard camera calibration parameters, especially in low to medium precision photogrammetric applications [8]. For example, fixing the zoom and focus rings with tape or epoxy, and turning off any product features, which counteract photogrammetric uses, such as auto focus, sensor filter vibration for dust removal, sensor or lens movement for vibration reduction/image stabilization [8].

In the case when photo variant orientation is not intended, and thus block invariant IOPs are assumed, it is necessary to assess the stability of the internal geometry of the camera in question. In other words, IOPs estimated from different calibration sessions must be checked for compatibility. This process is referred to as camera stability analysis. The level of variation found during the camera stability analysis can also serve as a guideline for the frequency of any necessary re-calibrations [12]. One way of assessing the stability of camera geometry is through statistical testing. The hypothesis whether the estimated values for an IOP parameter from two different calibration sessions are equivalent is either accepted or rejected based on the “change significance” test statistic shown in Equation (1) [12]:

(1) y=|xi+1−xi|σxi+12+σxi2 ~ Student's t

where *x* is the IOP parameter in question; σ^2^ is the variance associated with the parameter; and *i* and *i* + 1 are the two calibration sessions. Alternatively, instead of performing the test on individual parameters, *x*, it can be done on all the IOP parameters as a set, *x̂*, (see Equation (2)) [13–15]:

(2) $$y={\left({\hat{x}}_{i+1}-{\hat{x}}_{i}\right)}^{T}{\left(C_{x_{i+1}}+C_{x_{i}}\right)}^{-1}\left({\hat{x}}_{i+1}-{\hat{x}}_{i}\right)\sim \chi_{1-\alpha ,d}^{2}$$

where *C_x_* represents the variance-covariance matrix for a parameter set from a specific calibration session; and the critical value comes from a chi-squared distribution, *χ*^2^, at *α* significance level and with *d* degrees of freedom. The significance level, which is the probability of rejecting a true null hypothesis, is usually selected as 0.05, and the number of degrees of freedom equals the rank of the variance-covariance matrix or the number of parameters. This approach for stability analysis has the following drawbacks [13]:

- It is assumed that the estimated parameters are normally distributed and possess no biases;
- The variances for the estimated individual parameters or the variance-covariance matrices for the estimated parameter sets must be available; if variance-covariance matrices are not used, any potential correlations between the parameters would not be considered;
- It does not take into consideration any possible correlations between the IOPs and the exterior orientation parameters (EOPs); and
- Regardless of the outcome of the statistical test, the effect of the differences in the estimated parameters cannot be quantified in terms of quality of the reconstructed object space or image coordinate precision.

Thus, a measure of the equivalency for the IOPs in terms of their impact on the outcome from photogrammetric reconstruction (e.g., discrepancies in the object space coordinates or image space residuals) must be estimated separately in addition to the statistical test above. For example, Shortis *et al.* [16] reported an analysis of camera stability by using the ratio of mean precision of target coordinates to the largest dimension of the target array. This is because any unmodelled IOP errors may cause higher image space residuals (*i.e.*, poorer precision), and also higher root mean squared errors (RMSE) for known distances (*i.e.*, poorer accuracy) [17]. The down side of estimating a measure of equivalency for different sets of IOPs using real data and control information is that an object space test field or object space distances are needed for quality control purposes.

Habib and Morgan [13] and Habib *et al.* [14], however, performed camera stability analysis using simulation-based methods. The advantage of using simulated data is that there is no need for any additional control information. Moreover, their approach not only assessed the stability of the camera geometry, but at the same time, it also provided a measure of equivalency for the IOP sets in question. The aim of the authors actually was to evaluate the degree of similarity between the reconstructed light-ray bundles using two different sets of IOPs, derived from two different calibration sessions. This was achieved by computing the average offset between conjugate light rays within the simulated bundles along the image plane. This offset was compared to the expected image coordinate measurement precision in order to decide whether the two IOP sets were similar or not [13,14]. Three methods were introduced, and each one imposed constraints regarding the position and orientation of the defined bundles in space. Thus, each method proved to be applicable for a specific georeferencing methodology [18]. Lichti *et al.* [15] expanded on these methods by randomly simulating a large number of object space surfaces in order to decouple the stability assessment from the choice of terrain with a given height variation.

### 1.2. Stability Analysis of a Multi-Camera System

In the case of a single camera calibration, the parameters of interest are the IOPs. So the question in the stability analysis of a single camera is whether two sets of IOPs for a particular camera, estimated at different calibration sessions, are similar or not. When a photogrammetric system is comprised of multiple cameras, a system calibration must be performed. In this case of interest are not only the IOPs, but also the orientation parameters of each camera relative to a body frame or a reference camera. These relative orientation parameters are also sometimes referred to as the mounting parameters of the cameras to the platform they are attached to. So the stability analysis question for a multiple-camera system becomes whether the differences between two sets of IOPs and mounting parameters for the involved cameras, estimated at different calibration sessions, are significant or not. This would be referred to as system stability analysis in this paper.

While the stability analysis of a single camera has been addressed in literature, the stability analysis of a multi-camera photogrammetric system has only been touched on by one research group in relation to an underwater system for the measurement of sub-tidal organisms [19]. Their system consisted of two cameras attached on a base bar, with a check plate within the field of view of the cameras. Periodic measurements of the points on the check plate were used to detect any variability during a data acquisition session [20]. The platform/base bar orientation was taken as the average of the EOPs for the two cameras, and the mounting parameters were estimated as the “average” of the differential EOPs between the cameras from all the calibration exposures. The parameters were checked for stability based on the test for significance listed in Equation (1). In addition, the influence of the variations in the parameters on measured known lengths was estimated [21]. In another study by the same group, the stability of the system calibration parameters within a single deployment, and their stability over a series of deployments were investigated [22].

The statistical test used for checking significant changes between the calibration parameters of a multi-camera system suffers from the same drawbacks as in the case of a single camera. Also, having an object fixed to the system platform that is always visible within the overlapping fields of view of the cameras, or even simply using an object with known dimensions (*i.e.*, ground truth) may not be always possible. This paper addresses the issue of stability analysis of a photogrammetric system comprised of multiple cameras. The aim of this study is to first quantify the impact of different IOP and mounting parameter calibration sets on the reconstruction process for a pair of cameras expressed in image space units. Then, making the decision whether the two sets of system calibration parameters are compatible for quality object space reconstruction, would be based on the expected image measurement precision. Next section will describe the mathematical model used for a single-step system calibration. After that, three approaches for system stability analysis are presented, followed by the experimental results. At the end, conclusions and recommendations for future work are summarized.

## 2. System Calibration

A photogrammetric system includes one (moving) or multiple (stationary or moving together) digital cameras. If a single camera is used, it will have to sequentially occupy multiple camera stations. It should be noted that due to the time lapse between the multiple station exposures, this option would only work for stationary objects. Thus, multiple cameras should preferably be used in the scenarios where the objects or the platform are moving. Correct system calibration is essential for accurate object point determination [23]. This is especially crucial for direct sensor orientation in mobile mapping applications [24,25], dense image matching for full surface/object reconstruction [26,27], long-term infrastructure monitoring [28,29], biomedical and motion-capture metric applications [30–32], the generation of photo scenes from multiple sensors [33,34].

As mentioned before, the calibration parameters for a multi-camera photogrammetric system include the IOPs and the mounting parameters for each involved camera relative to a body frame or a reference camera. The IOPs should have been ideally estimated prior to the data collection campaign. However, in the case when the system consists of many cameras, and/or disassembling them from the platform is not desirable, the IOP estimation must be done *in-situ* or on-the-job. The challenge of such an IOP calibration approach is to guarantee adequate network geometry. For example, multiple-station convergent images with a good base-to-depth ratio and enough tie points with an even image-format distribution must be present for each camera. This network configuration can be simulated by translating and rotating a portable test field, while keeping the camera system in place. Assuming that the mounting parameters are defined to be relative to a reference camera, they consist of positional/baseline, *r* (the vector $r_{a}^{b}$ signifies a translation from *b* to *a* or the lever arm between *b* and *a* relative to the b-frame), and angular/rotational, *R* (the matrix $R_{a}^{b}$ should be read as the rotation matrix, which takes you from frame *a* to frame *b*) offsets between the different cameras and the reference one. These components can also be referred to as the lever arm and boresight, respectively. There exist two-step and one-step procedures for estimating the mounting parameters.

**Figure 2.**
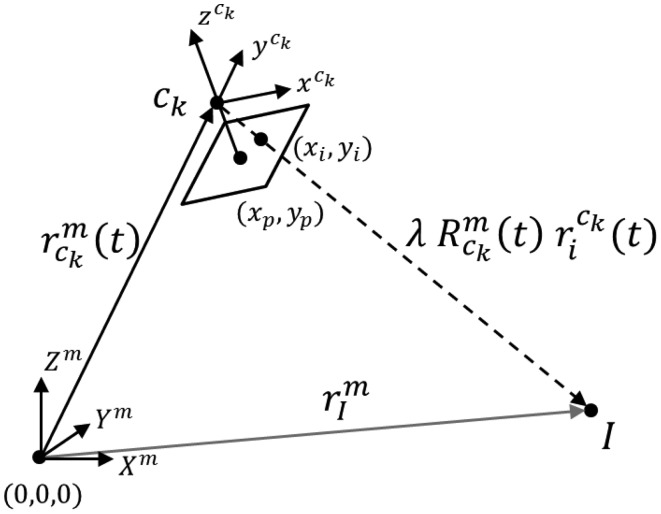
Visual description of the collinearity model used in a conventional bundle block adjustment.

The two-step procedure first estimates the EOPs for the different cameras through a conventional bundle block adjustment based on the collinearity equations (see Equations (3) and (4)). The collinearity model is also visually depicted in Figure 2.

(3) $$r_{I}^{m}=r_{c_{k}}^{m}\left(t\right)+\lambda R_{c_{k}}^{m}(t)r_{i}^{c_{k}}(t)$$

where $r_{I}^{m}$ is the position of the object point, *I*, with respect to the mapping frame *m*; $r_{c_{k}}^{m}\left(t\right)$ and $R_{c_{k}}^{m}\left(t\right)$ are the time-dependent positional and rotational parameters or the EOPs of camera *c_k_* with respect to the mapping frame *m*; *λ* is the scale; and the expression

(4) $$r_{i}^{c_{k}}\left(t\right)=\left[\begin{array}{c} x_{i}^{c_{k}}-x_{p}^{c_{k}}-\Delta x_{i}^{c_{k}}\\ y_{i}^{c_{k}}-y_{p}^{c_{k}}-\Delta y_{i}^{c_{k}}\\ -c^{c_{k}} \end{array}\right]$$

is the distortion free position of the image point, *i*, in the frame of camera *c_k_*, where (*x_i_*, *y_i_*) are the observed image coordinates for point *i*; (*x_p_*, *y_p_*) is the principal point offset; *c* is the principal distance; and (Δ*x_i_*, Δ*y_i_*) are the image space distortions for point *i*.

The mounting parameters are then derived from the EOPs using Equations (5) and (6) [25]:

(5) $$r_{c_{k}}^{c_{r}}\left(t\right)={\left(R_{c_{r}}^{m}(t)\right)}^{-1}(r_{c_{k}}^{m}(t)-r_{c_{r}}^{m}(t))$$

where $r_{c_{k}}^{c_{r}}\left(t\right)$ is the time-dependant lever arm/positional offset between camera *c_k_* and the reference camera *c_r_*; $r_{c_{r}}^{m}\left(t\right)$ and $R_{c_{r}}^{m}(t)$ are the time-dependant positional and rotational parameters or the EOPs of the reference camera *c_r_* with respect to the mapping frame *m*; and $r_{c_{k}}^{m}(t)$ is the time-dependant positional portion of the EOPs of camera *c_k_*;

(6) $$R_{c_{k}}^{c_{r}}\left(t\right)={\left(R_{c_{r}}^{m}(t)\right)}^{-1}R_{c_{k}}^{m}(t)$$

where $R_{c_{k}}^{c_{r}}\left(t\right)$ is the time-dependant boresight/rotational offset between camera *c_k_* and the reference camera *c_r_*; and $R_{c_{k}}^{m}(t)$ is the time-dependant rotational portion of the EOPs of camera *c_k_*. At the end, the resultant time-dependant mounting parameters can be averaged, and their standard deviations can be computed [25].

The one step procedure is usually based on constrained equations, which are used to enforce an invariant geometrical relationship between the cameras at different times [33,35–39]. For example, if the number of cameras involved in the system is *n_k_*, and the number of observation epochs is *c_t_*, then the total number of EOPs is 6*n_k_n_t_*. Assuming that the lever arm and boresight components are not changing over time, the number of constraints that can be introduced is 6(*n_k_* – 1) (*n_t_* – 1) as seen in Equations (7) and (8). So the number of independent parameters that define the EOPs of the different cameras at all the observation epochs is 6(*n_k_* – 1) + 6*n_t_*:

(7) $$\begin{array}{c} r_{c_{2}}^{c_{1}}\left(t_{1}\right)=r_{c_{2}}^{c_{1}}\left(t_{2}\right)=\ldots =r_{c_{2}}^{c_{1}}\left(t_{n_{t}}\right)\\ r_{c_{3}}^{c_{1}}\left(t_{1}\right)=r_{c_{3}}^{c_{1}}\left(t_{2}\right)=\ldots =r_{c_{3}}^{c_{1}}\left(t_{n_{t}}\right)\\ \ldots \\ r_{c_{n_{k}}}^{c_{1}}\left(t_{1}\right)=r_{c_{n_{k}}}^{c_{1}}\left(t_{2}\right)=\ldots =r_{c_{n_{k}}}^{c_{1}}\left(t_{n_{t}}\right)\\ =>3\left(n_{k}-1\right)\left(n_{t}-1\right)\text{constraints} \end{array}$$

(8) $$\begin{array}{c} R_{c_{2}}^{c_{1}}\left(t_{1}\right)=R_{c_{2}}^{c_{1}}\left(t_{2}\right)=\ldots =R_{c_{2}}^{c_{1}}\left(t_{n_{t}}\right)\\ R_{c_{3}}^{c_{1}}\left(t_{1}\right)=R_{c_{3}}^{c_{1}}\left(t_{2}\right)=\ldots =R_{c_{3}}^{c_{1}}\left(t_{n_{t}}\right)\\ \ldots \\ R_{c_{n_{k}}}^{c_{1}}\left(t_{1}\right)=R_{c_{n_{k}}}^{c_{1}}\left(t_{2}\right)=\ldots =R_{c_{n_{k}}}^{c_{1}}\left(t_{n_{t}}\right)\\ =>3\left(n_{k}-1\right)\left(n_{t}-1\right)\text{constraints} \end{array}$$

The downside of using such relative orientation constraints is that the complexity of the implementation procedure intensifies with the increase of the number of cameras in the system and the number of observation epochs [25]. The one step procedure used here directly incorporates the relative orientation constraints among all cameras and the body frame/reference camera in the collinearity equations as seen in Equation (9) [25,35,36]:

(9) $$r_{I}^{m}=r_{c_{r}}^{m}\left(t\right)+R_{c_{r}}^{m}(t)r_{c_{k}}^{c_{r}}+\lambda R_{c_{r}}^{m}(t)R_{c_{k}}^{c_{r}}r_{i}^{c_{k}}(t)$$

**Figure 3.**
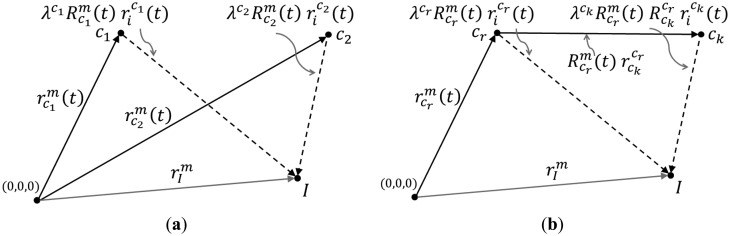
Mathematical model for 3D reconstruction using separate EOPs for each camera station (**a**) *versus* using EOPs for a reference camera and ROPs for the rest of the cameras (**b**).

The mounting parameters, $r_{c_{k}}^{c_{r}}$ and $R_{c_{k}}^{c_{r}}$, are now time-independent, and the EOPs of the reference camera, $r_{c_{r}}^{m}\left(t\right)$ and $R_{c_{r}}^{m}(t)$, now represent the EOPs of the system platform. This model preserves its simplicity regardless of the number of cameras or the number of observation epochs. It should be noted that instead of solving for 6*n_k_n_t_* EOP unknowns, the adjustment will solve for 6*n_t_* EOPs for the reference camera in addition to 6(*n_k_* – 1) mounting parameters for the rest of the cameras with respect to the reference camera. This is equivalent to the total number of independent parameters that are needed to represent the EOPs of the different cameras at all the data acquisition epochs. The reduction in the number of parameters to solve for in the adjustment will also reduce any possible high correlations between the many system calibration parameters. The difference in the bundle adjustment mathematical models described in Equations (3) and (9) is visually summarized in Figure 3.

## 3. System Stability Analysis

In this section, three methodologies will be presented that simultaneously compare two IOP sets, *IOP_i_*(*t*_1_) and *IOP_j_*(*t*_1_) with *IOP_i_*(*t*_2_) and *IOP_j_*(*t*_2_), and two mounting parameter sets, $r_{c_{j}}^{c_{i}}(t_{1})$ and $R_{c_{j}}^{c_{i}}(t_{1})$ with $r_{c_{j}}^{c_{i}}(t_{2})$ and $R_{c_{j}}^{c_{i}}(t_{2})$, for two camera stations contributing to the 3D reconstruction, *c_i_* and *c_j_*, derived from two calibration sessions, *t*_1_ and *t*_2_. Since the mounting parameters output from a system calibration at *t* is actually $r_{c_{i}}^{c_{r}}(t)$, $R_{c_{i}}^{c_{r}}(t)$, $r_{c_{j}}^{c_{r}}(t)$ and $R_{c_{j}}^{c_{r}}(t)$, the desired mounting parameters for comparison are computed using Equations (10) and (11):

(10) $$r_{c_{j}}^{c_{i}}\left(t\right)={\left(R_{c_{i}}^{c_{r}}(t)\right)}^{-1}(r_{c_{j}}^{c_{r}}(t)-r_{c_{i}}^{c_{r}}(t))$$

(11) $$R_{c_{j}}^{c_{i}}\left(t\right)={\left(R_{c_{i}}^{c_{r}}(t)\right)}^{-1}R_{c_{j}}^{c_{r}}(t)$$

**Figure 4.**
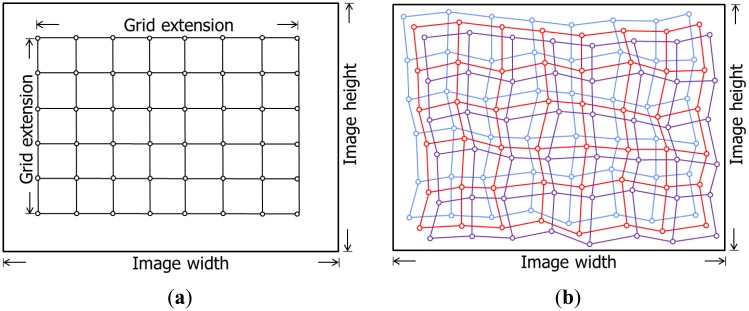
Simulated grid of image points in the format of one camera (**a**); the projection of the simulated points in the image format of the other camera using different object space depths (**b**).

**Figure 5.**
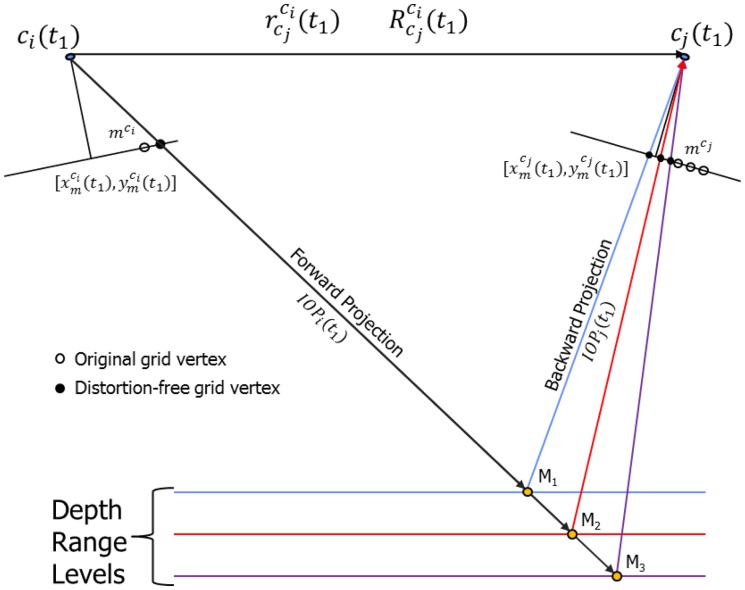
Example of the original and distortion-free grid vertices used in the forward and backward projections at different object space depths.

The objective is to decide whether the cumulative effect of the two sets of IOPs and mounting parameters on the reconstruction process is equivalent or not. In other words, for a given image dataset, does the reconstruction outcome depend on using either set of system calibration parameters? If the 3D reconstruction principle is based on pairwise matching and tracking of conjugate features, then the system stability analysis should be run on every consecutive camera pair. However, if exhaustive matching is performed, then the system stability analysis must be run on every overlapping camera pair. As with the stability analysis for a single camera, the proposed methodologies for system stability analysis are simulation-based (note that the term “simulation-based” only refers to the fact that a synthetic grid in image space is used for evaluating the stability of the system parameters; the system parameters being tested are real, not simulated). The proposed methodologies have the following structure:

- Define a synthetic regular grid in the image space of one of the cameras, *c_i_* (see Figure 4a);
- Use the IOPs and mounting parameters of this camera from the first calibration session to remove the distortions at the grid vertices and compute the object space coordinates of each vertex by forward projecting them to a range of plausible object space depths (see Figure 5);
- Compute the image space coordinates of the grid points for the other camera, *c_j_*, by backward projection using the IOPs and mounting parameters for the other camera from the first calibration session (see Figure 5). Note that the different depth values will yield multiple “grids” in the image space of the other camera (see Figure 4b);
- Estimate the effect of having different IOPs and mounting parameters from another calibration session in image units for all simulated points and all depth levels using one of the proposed methodologies, which will be introduced later; and
- Compare the RMSE value for all the differences/offsets to the expected image space coordinate measurement precision; if the RMSE value is the smaller one, then the system is deemed stable, and if the RMSE value is the greater one, the system would be considered unstable.

The proposed methodologies for the system stability analysis are: (1) combination of forward and backward projections; (2) object space parallax in image units; and (3) variation in the normalized image coordinates. They are explained in the next three sub-sections.

### 3.1. Method 1: Combination of Forward and Backward Projections

**Figure 6.**
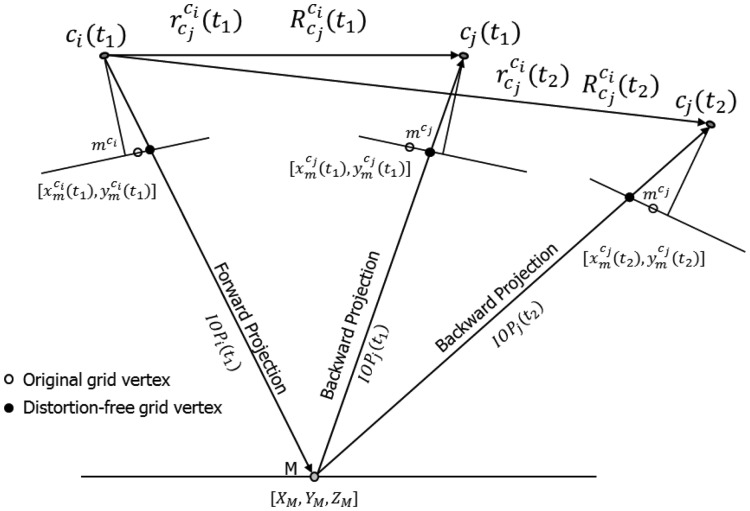
Illustration of the combination of forward and backward projections methodology.

This methodology aims at reporting the displacement in the second image of a stereo pair. The displacement would be due to the changes in the IOPs and mounting parameters relating the two camera stations from one epoch to another. The grid of points from one camera, *c_i_*, is first forward projected to the object space with one set of system calibration parameters. The object space coordinates are then backward projected to the image space of the other camera, *c_j_*, using the two different sets of system calibration parameters (see Figure 6).

Having estimated the corresponding image coordinates for epochs *t*_1_ and *t*_2_ in camera *c_j_* for all grid vertices and for all depth ranges, the RMSE values for the *x* and *y* components can be computed based on the differences shown in Equations (12) and (13):

(12) $$\delta x_{m}=x_{m}^{c_{j}}\left(t_{1}\right)-x_{m}^{c_{j}}\left(t_{2}\right)$$

(13) $$\delta y_{m}=y_{m}^{c_{j}}\left(t_{1}\right)-y_{m}^{c_{j}}\left(t_{2}\right)$$

It should be noted that change in the IOPs for the first camera is not considered during the system stability analysis, but it can be checked separately via a single camera stability analysis (e.g., using one of the approaches listed in Habib *et al.* [18] or Lichti *et al.* [15]). Alternatively, this procedure could be repeated a second time, where the roles of cameras *c_i_* and *c_j_* are reversed. Then, the RMSE values, averaged from the two runs, could be used as the final measure.

### 3.2. Method 2: Object Space Parallax in Image Units

When it comes to 3D reconstruction, the extent of the x-parallax between conjugate points in overlapping images is what defines the object shape or the camera to object depth. The x-parallax can be defined either as an image space or object space displacement component, usually along the baseline connecting the two perspective centres of a stereo-pair, between conjugate image points or their projection onto a given object space plane. On the other hand, the image matching process will be more difficult, and the 3D reconstruction would be less precise, without removing the y-parallax between conjugate points in overlapping images. The y-parallax can be defined either as an image space or object space displacement component, usually along the perpendicular to the baseline, between conjugate image points or their projection onto a given object space plane. So studying how the changes in the system calibration parameters would impact the x- and y-parallax should be considered essential for the system stability analysis. In this methodology, the x- and y-parallax are first evaluated by quantifying the object space discrepancy arising from the variations in the IOPs and mounting parameters for both cameras. This discrepancy is decomposed into x- and y-components, which are then scaled to image units.

**Figure 7.**
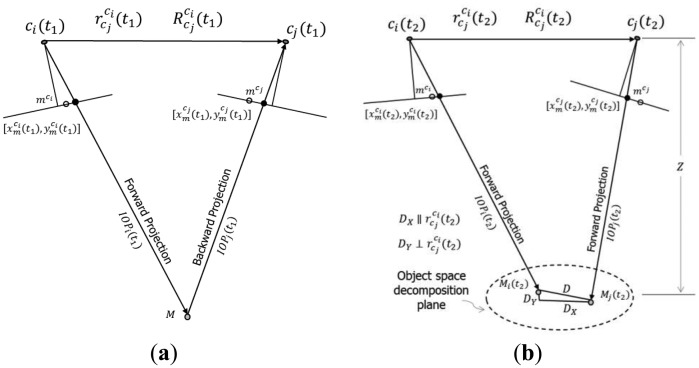
Example of the object space parallax in image units methodology: forward and backward projections for the first epoch (**a**); forward projections for the second epoch (**b**).

In this approach, the grid vertices in one of the cameras, *c_i_*, are first forward projected to object space at a certain depth range using the IOPs and mounting parameters for the first calibration session. The resultant object space points are then backward projected to the image space of the other camera, *c_j_* (see Figure 7a). Once the object space and the image space coordinates are established using the first set of system calibration parameters, the scale factor *λ* for one of the cameras can be computed after rearranging the terms in Equation (9). Next, the image space coordinates for this camera are forward projected while preserving the same scale factor, but this time using the IOPs and mounting parameters from the second calibration epoch. An object space plane, which will later be denoted as the object space decomposition plane, is then generated through the resultant object space point. This object space plane fulfills two conditions: (1) it is parallel to the baseline; and (2) its roll relative to the baseline equals the average roll of both cameras. The image space coordinates for the other camera, *c_j_*, can now be also forward projected to the generated object space plane using the second set of IOPs and mounting parameters. The resultant object space parallax, or discrepancy vector between the projected points from cameras *c_i_* and *c_j_*, is decomposed into two components within the generated object space plane. The first component, *D_X_*, is parallel to the baseline, while the second component, *D_Y_*, is perpendicular to the baseline (see Figure 7b). The two object space parallax components can then be converted to image units by using the ratio between the average principal distance, *c_avg_* = (*c^c_i_^* + *c^c_j_^*)/2, and the object space depth, *Z*, *i.e.*, the normal distance from the baseline to the object space decomposition plane (see Equations (14) and (15)). The RMSE for *δx* and *δy* are again based on all the grid vertices and all the depth levels. One should note that the outcome from this method does not depend on the choice of the scale factor for the forward projection for the second epoch since the object space discrepancy is ultimately scaled back to image units. However, the scale factor would be only useful for defining realistic values for the object space parallax values, *D_X_* and *D_Y_*.

(14) $$\delta x=D_{X}\frac{c_{\text{avg}}}{Z}$$

(15) $$\delta y=D_{Y}\frac{c_{\text{avg}}}{Z}$$

### 3.3. Method 3: Variation in the Normalized Image Coordinates

**Figure 8.**
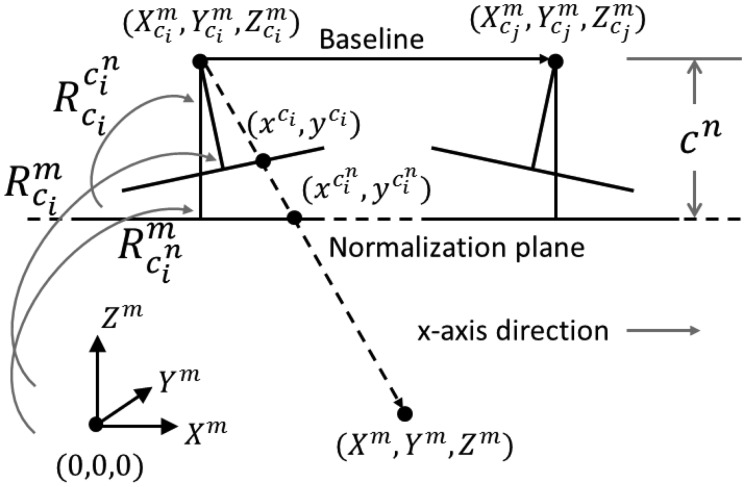
Example of normalized images generation.

Rather than evaluating the object space discrepancy as a result of changes in the system calibration parameters, and then scaling them down to the image space, this approach directly evaluates the image space impact. Method 3 achieves this through the concept of normalized image generation or the generation of images normalized according to epipolar geometry. Normalized images are synthesized stereo images, which share the same perspective centre with the original images, but their image planes are parallel to the baseline between the two cameras (with the x-axes being parallel to the baseline) (see Figure 8). Within the synthesized images, given that accurate IOPs and mounting parameters from a valid system calibration are used, the x-parallax between conjugate points is proportional to the depth, and there is no y-parallax between those points. In this approach, the normalized image coordinates for the stereo pair *c_i_* and *c_j_* are generated using the first set of system calibration parameters. Then, the impact of the variations in the IOPs and mounting parameters on the normalized image coordinates is studied.

As seen in Figure 8, the coordinates of an object space point relative to the perspective centre of an image from a particular camera can be expressed as the scaled vector from the perspective centre to the original image point in the frame for that camera, *c_k_*, after transforming it to the object space/mapping reference frame (see Equation (16)).

(16) $$\left[\begin{array}{c} X^{m}-X_{c_{k}}^{m}\\ Y^{m}-Y_{c_{k}}^{m}\\ Z^{m}-Z_{c_{k}}^{m} \end{array}\right]=\lambda^{c_{k}}R_{c_{k}}^{m}\left[\begin{array}{c} x^{c_{k}}-x_{p}^{c_{k}}-\Delta x^{c_{k}}\\ y^{c_{k}}-y_{p}^{c_{k}}-\Delta y^{c_{k}}\\ -c^{c_{k}} \end{array}\right]$$

where the rotation matrix $R_{c_{k}}^{m}$ is based on the original attitude angles for the camera $\omega_{c_{k}}^{m}$, $\varphi_{c_{k}}^{m}$, and $\kappa_{c_{k}}^{m}$. Alternatively, the coordinates of an object space point relative to the perspective centre of an image from a particular camera can be expressed as the scaled vector from the perspective centre to the normalized image point in the normalized image frame for that camera, $c_{k}^{n}$, after transforming it to the object space/mapping reference frame (see Equation (17)):

(17) $$\left[\begin{array}{c} X^{m}-X_{c_{k}}^{m}\\ Y^{m}-Y_{c_{k}}^{m}\\ Z^{m}-Z_{c_{k}}^{m} \end{array}\right]=\lambda^{c_{k}^{n}}R_{c_{k}^{n}}^{m}\left[\begin{array}{c} x^{c_{k}^{n}}\\ y^{c_{k}^{n}}\\ -c^{n} \end{array}\right]$$

where the rotation matrix $R_{c_{k}^{n}}^{m}$, which depends on the components of the baseline between the two cameras, is based on the normalized attitude angles $\omega_{c_{k}^{n}}^{m}$, $\varphi_{c_{k}^{n}}^{m}$, and $\kappa_{c_{k}^{n}}^{m}$. These attitude angles are needed to make the image plane parallel to the baseline and the x-axis of the normalized image parallel to the baseline [40]. After equating the right hand sides of Equations (16) and (17), and isolating the normalized image coordinates, Equation (18) could be derived:

(18) $$\left[\begin{array}{c} x^{c_{k}^{n}}\\ y^{c_{k}^{n}}\\ -c^{n} \end{array}\right]=\frac{\lambda^{c_{k}}}{\lambda^{c_{k}^{n}}}R_{c_{k}}^{c_{k}^{n}}\left[\begin{array}{c} x^{c_{k}}-x_{p}^{c_{k}}-\Delta x^{c_{k}}\\ y^{c_{k}}-y_{p}^{c_{k}}-\Delta y^{c_{k}}\\ -c^{c_{k}} \end{array}\right]$$

where

(19) $$R_{c_{k}}^{c_{k}^{n}}=R_{m}^{c_{k}^{n}}R_{c_{k}}^{m}$$

The rotation matrix $R_{c_{k}}^{c_{k}^{n}}$ is based on the angles $\omega_{c_{k}}^{c_{k}^{n}}$, $\varphi_{c_{k}}^{c_{k}^{n}}$, and $\kappa_{c_{k}}^{c_{k}^{n}}$, and is used to transform the image coordinates from the original image frame to the normalized image frame [40]. It should be noted again that the rotation matrix $R_{m}^{c_{k}^{n}}$ depends on the baseline components, *i.e.*, $r_{c_{j}}^{c_{i}}$. Thus, indirectly, the rotation matrix $R_{c_{k}}^{c_{k}^{n}}$ also depends on the baseline components. It should also be noted that the mapping frame, *m*, can be chosen to be the coordinate system of camera *c_i_*, while *c_k_* would correspond to *c_i_* and *c_j_* when generating the normalized image coordinates for cameras *c_i_* and *c_j_*, respectively.

All in all, as presented in Equations (20) and (21), the computation of normalized image coordinates is a function of the IOPs and mounting parameters for an image pair:

(20) $$x^{c_{k}^{n}}=f_{x}(x,y,IOPs,\;\omega_{c_{k}}^{c_{k}^{n}},\varphi_{c_{k}}^{c_{k}^{n}},\kappa_{c_{k}}^{c_{k}^{n}})$$

(21) $$y^{c_{k}^{n}}=f_{y}(x,y,IOPs,\;\omega_{c_{k}}^{c_{k}^{n}},\varphi_{c_{k}}^{c_{k}^{n}},\kappa_{c_{k}}^{c_{k}^{n}})$$

So the variations in the IOPs and the mounting parameters from the two sets of system calibration parameters can be used to estimate the resulting changes in the normalized image coordinates as per Equations (22) and (23):

(22) $$\delta x^{c_{k}^{n}}=\;\frac{\partial f_{x}}{\partial IOPs}\delta IOPs+\frac{\partial f_{x}}{\partial \omega_{c_{k}}^{c_{k}^{n}}}\delta \omega_{c_{k}}^{c_{k}^{n}}+\frac{\partial f_{x}}{\partial \varphi_{c_{k}}^{c_{k}^{n}}}\delta \varphi_{c_{k}}^{c_{k}^{n}}+\frac{\partial f_{x}}{\partial \kappa_{c_{k}}^{c_{k}^{n}}}\delta \kappa_{c_{k}}^{c_{k}^{n}}$$

(23) $$\delta y^{c_{k}^{n}}=\;\frac{\partial f_{y}}{\partial IOPs}\delta IOPs+\frac{\partial f_{y}}{\partial \omega_{c_{k}}^{c_{k}^{n}}}\delta \omega_{c_{k}}^{c_{k}^{n}}+\frac{\partial f_{y}}{\partial \varphi_{c_{k}}^{c_{k}^{n}}}\delta \varphi_{c_{k}}^{c_{k}^{n}}+\frac{\partial f_{y}}{\partial \kappa_{c_{k}}^{c_{k}^{n}}}\delta \kappa_{c_{k}}^{c_{k}^{n}}$$

where the differences in the IOPs, *δIOPs*, are simply *(IOPs*(*t*_2_) – *IOPs*(*t*_2_)), and $\delta \omega_{c_{k}}^{c_{k}^{n}}$, $\delta \varphi_{c_{k}}^{c_{k}^{n}}$, and $\delta \kappa_{c_{k}}^{c_{k}^{n}}$ are extracted from the combined rotation matrix in Equation (24):

(24) $$R_{c_{k}^{n}(t_{2})}^{c_{k}^{n}(t_{1})}=R_{c_{k}}^{c_{k}^{n}}(t_{1})R_{c_{k}^{n}}^{c_{k}}(t_{2})$$

The *x* and *y* RMSE values for this method are based on the changes in the *x*- and *y*-parallax values for the normalized image coordinates shown in Equations (25) and (26) for all the grid vertices and all the depth levels:

(25) $$\delta p_{x^{n}}=p_{x^{n}}\left(t_{2}\right)-p_{x^{n}}\left(t_{1}\right)=\;\delta x^{c_{i}^{n}}-\delta x^{c_{j}^{n}}$$

(26) $$\delta p_{y^{n}}=p_{y^{n}}\left(t_{2}\right)-p_{y^{n}}\left(t_{1}\right)=\;\delta y^{c_{i}^{n}}-\delta y^{c_{j}^{n}}$$

**Figure 9.**
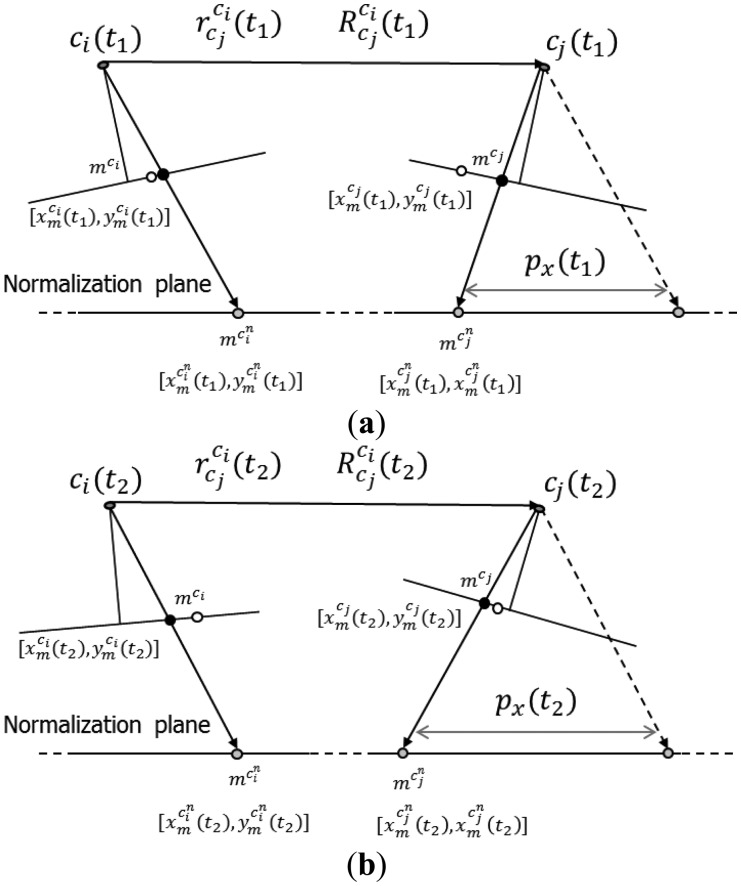
Side view for the image coordinate normalization at one epoch (**a**); and changes in the imaging geometry, the normalized image coordinates, and the resultant x-parallax at another epoch (**b**).

A large value for the change in the parallax in the x-direction, $\delta p_{x^{n}}$, means that the shape of a reconstructed object would significantly differ depending on whether the IOP and mounting parameters from calibration epoch one or calibration epoch two are used. Figure 9 shows a side view example of the image coordinate normalization using the IOPs and mounting parameters derived from two different calibration sessions with emphasis on the change in the x-parallax value, $p_{x^{n}}$.

**Figure 10.**
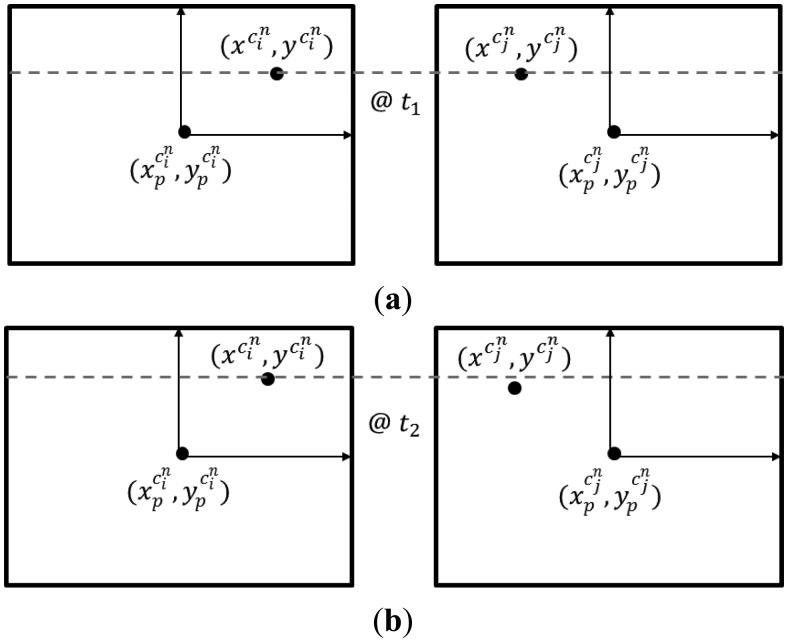
Top view for the image coordinate normalization at one epoch with emphasis on conjugate points being on the same image row (**a**); and changes in the normalized image coordinates at another epoch with emphasis on conjugate points not necessary being on the same image row any more (**b**).

Similarly, a large value for the change in the parallax in the y-direction, $\delta p_{y^{n}}$, would mean that the image matching of conjugate points will be negatively affected if the system calibration parameters are not stable over time. Figure 10 shows the top view for the example shown in Figure 9. Here, provided that the system calibration at epoch one was successful, the y-coordinates in the normalized image spaces for a conjugate point should be equal. If there is significant changes in the y-normalized image coordinates due to variations in the IOPs and mounting parameters for the two cameras, the y-coordinates for the conjugate points at epoch two would not necessarily be equal.

### 3.4. Discussion of the Proposed Methodologies

As mentioned earlier, Method 1, combination of forward and backward projections, does not consider the variation in the IOPs of the first camera when deriving the stability analysis measure. Ignoring such a variation would be acceptable as long as the system instability is mainly assumed to arise from changes in the mounting parameters (*i.e.*, lever arm components and boresight angles) relating the camera stereo pairs.

The implementation of Method 2, object space parallax in image units, is slightly more complex when compared to Method 1. However, this approach comprehensively considers the variations in the IOPs of both cameras in a stereo pair as well as any changes in the mounting parameters relating the two camera stations.

**Figure 11.**
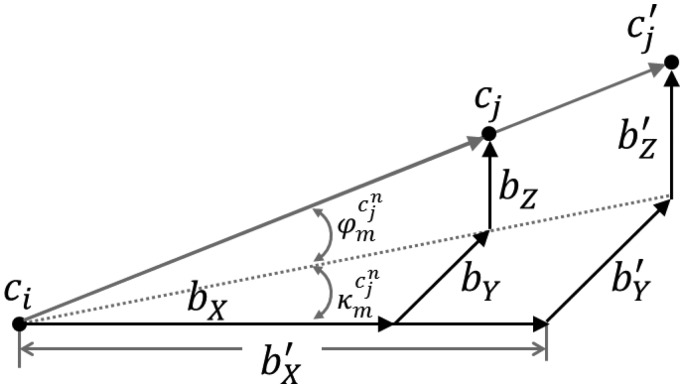
Example of changes in the magnitude of the baseline between two cameras while maintaining its orientation.

Method 3, variation in the normalized image coordinates, can be considered to be slightly simpler (*i.e.*, easier to grasp) when compared to the previous approach. Similarly to Method 2, this method considers both variations in the IOPs of the involved cameras together with any changes in the mounting parameters relating the two camera stations. However, closer examination of this approach would reveal that the variations in the mounting parameters between the two calibration sessions are not fully considered. More specifically, while considering variations in the rotational relationship and the orientation of the baseline between the two camera stations, this approach does not consider changes in the magnitude (*i.e.*, the extent) of the baseline. As can be seen in Figure 11, changing the extent of the baseline between the two camera stations while preserving its orientation in space would not lead to any changes in the rotation matrix $R_{c_{k}^{n}}^{m}$, which in turn will not lead to any incremental changes in $\omega_{c_{k}}^{c_{k}^{n}}$, $\varphi_{c_{k}}^{c_{k}^{n}}$, and $\kappa_{c_{k}}^{c_{k}^{n}}$. In other words, changing the magnitude of the base line would not cause any changes in the estimated $\delta p_{x^{n}}$ and $\delta p_{y^{n}}$ values. One can conclude that changing the orientation of the baseline would impact the y-parallax between conjugate points, while changes in the magnitude of the baseline will only affect the scale of the reconstructed object, which in turn will impact the x-parallax between conjugate points. Therefore, this approach would only give a realistic estimate of the impact of variations in the system calibration parameters on the y-parallax or the precision of the reconstruction process. However, it will not provide accurate evaluation of the impact on the x-parallax or the shape (*i.e.*, scale) of the reconstructed object space. Therefore, if we are mainly concerned with the ability to have a precise 3D reconstruction of the object in question, this approach would still be valid.

In summary, if insignificant changes are expected for both the IOPs of the first camera as well as the extent of the lever arm between the two camera stations, one could argue that the three approaches are expected to have similar results. Also, provided that a system is stable, any set of IOPs and mounting parameters will produce a compatible object space for a given real image data of the object of interest. The experimental results in the next section will attempt to verify these hypotheses.

## 4. Experimental Results

**Figure 12.**
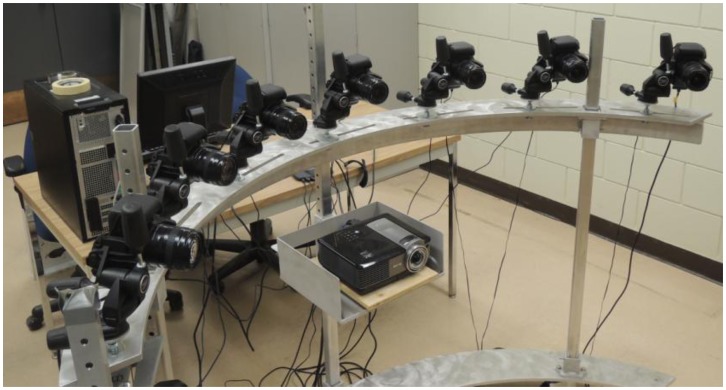
Multi-camera photogrammetric system consisting of seven DSLRs attached to a metal frame.

In order to test the proposed methods for system stability analysis, a multi-camera photogrammetric system was set up and calibrated three times. The system is comprised of seven digital single-lens reflex (DSLR) cameras, namely Canon EOS 1100D/Rebel T3 units. This model has a 22.2 × 14.7 mm^2^ complementary metal-oxide semiconductor (CMOS) sensor divided into 4272 × 2848 or 12.2 mega pixels, with each pixel having 5.2 μm pixel size in both dimensions. The focal length of the lenses was set to the nominal value of 30 mm. Image stabilization was turned off. The focus and shooting modes were switched to manual. Both the zoom and focus rings were also physically fixed with electrical tape. It should be noted that this camera model does not have an automatic sensor cleaning or dust shaking function. The cameras were attached to tripod heads with three degrees of freedom, which were mounted to a curved metal frame (see Figure 12). Figure 12 also shows a digital projector, which was used to project artificial texture on the object of interest in order to aid the 3D surface reconstruction performed later on.

**Figure 13.**
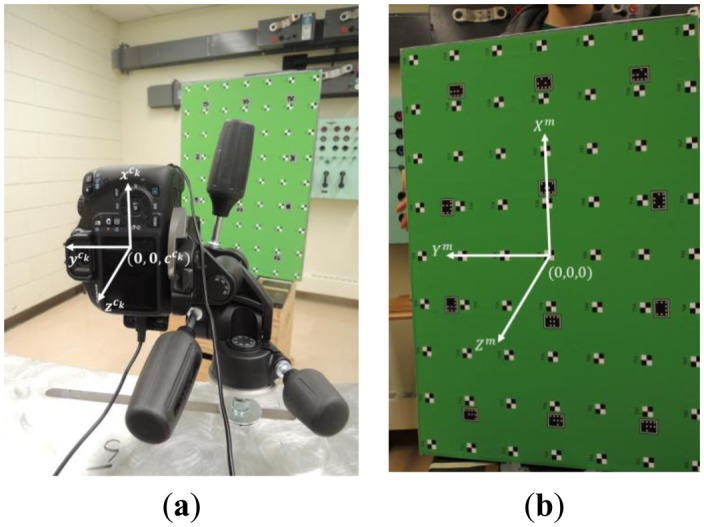
Example of the coordinate system used for a particular camera (**a**); calibration test field, and the origin and orientation of the local coordinate system (**b**).

In order for the image format to be proportional to the dimensions of the object of interest the system was designed for, each camera was oriented with the x-axis of its coordinate system pointing upwards, *i.e.*, in portrait as opposed to landscape mode (see Figure 13a). This meant that for this particular setup, the y-direction was the one parallel, and the x-direction was the one perpendicular to the baseline. The baseline between neighbouring cameras was approximately 0.3 m, while the distance from the cameras to where the object of interest would be placed was about 1 m on average. The camera station network had convergent geometry with the most outside cameras being at nearly 90° from each other.

The cameras were operated through a software package installed on a desktop computer. The software was used to control the camera settings, synchronize the cameras, and download the images to the computer hard drive. The communication link between the cameras and the computer was established through universal serial bus (USB) cables and hub.

**Figure 14.**
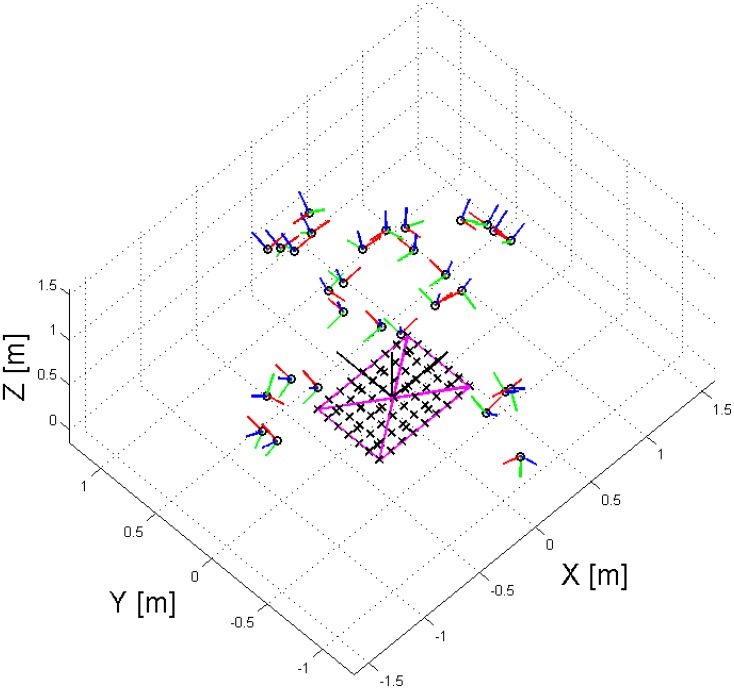
3D view for the positions and orientations of one of the cameras with respect to the test field; the camera stations are shown as circles, the camera x-axis is in red, the y-axis in green, and the z-axis in blue; the checkerboard targets are shown as crosses, and the magenta lines indicate distance measurements observed with a steel tape.

The test field used was a 2D board with a seven by nine grid of checkerboard targets and twelve coded targets. The origin of the local coordinate system was at the central checkerboard target, and the coded targets were used for automating the target labelling/correspondence problem (see Figure 13b). The coordinates of the checkerboard targets were used as unknowns in the adjustment, except for six coordinates, which were fixed in order to define a minimally constrained datum. In order to avoid projective compensation between the interior and exterior orientation parameters, convergent geometry and a roll of the test field was implemented. At the horizontal/landscape orientation, the *ω* rotation varied from −70° to +70°, while the *φ* tilt varied from −12.5° to +12.5°. At the vertical/portrait orientation, it was the other way around—the *φ* rotation varied from −70° to +70°, while the *ω* tilt varied from −12.5° to +12.5°. This amounted to a total of more than 160 images, from up to 30 observation epochs per camera. Figure 14 shows the positions and orientations of one of the cameras with respect to the test field.

The described data collection scheme was repeated for three calibration sessions. An in-house bundle adjustment with self-calibration, which was able to handle the mathematical model introduced in Equation (9), was used to produce a set of system calibration parameters for each calibration session. The central camera, *i.e.*, Camera 4, was used as the reference camera in all three calibration sessions. The IOP results from the multi-system calibration are shown in Table 1. It could be seen that the principal point offset and the principal distance parameters were solved with standard deviations of a few microns, and that the standard deviations for the radial lens distortion parameters are one to two orders of magnitude smaller than the actual parameter values.

The mounting parameter results are shown in Table 2. It could be seen that the lever arm parameters were solved with standard deviations of 0.03 to 0.3 mm, while the boresight parameters had standard deviations in the range of 3 to 24 arc s. Also, in all three calibration session results, the final sigma value was 1.8 μm, or about 1/3 of the pixel size, which was deemed satisfactory.

The proposed methods for system stability analysis were run with the resultant system calibration parameters. Each of the three calibration sessions were compared to the other two with the camera pairs of interest being the six consecutive camera pairs in the system, *i.e.*, cameras 1 & 2, 2 & 3, 3 & 4, 4 & 5, 5 & 6, and 6 & 7 (see Table 3). From the results for the system stability analysis, it could be seen that the total RMSE values ranged between 0.21–2.04 pixels with the average being 0.82 pixels for Method 1, 0.12–0.56 pixels with an average of 0.30 for Method 2, and 0.08–1.24 pixels and an average of 0.63 pixels for Method 3. In the case of Method 2, all of the stereo-pair total RMSE values were well under one pixel. In fact, they closely approximated the final sigma value reported from the system calibration bundle adjustments for the three calibration sessions. In the case of the other two methods, there was only one stereo-pair (*i.e.*, Cameras 5 & 6 for Method 1, and Cameras 6 & 7 for Method 3) with a total RMSE value over one pixel. As mentioned in the methodology discussion section, the first method does not consider any changes in the IOPs for one of the cameras, and the third method does not consider any changes in the extent of the baseline between the two cameras. Note that, in Table 3, the larger RMSE values for Method 3 are predominantly in the component parallel to the baseline.

Thus, Method 2 would be the one recommended for performing system stability analysis. However, in the case when the expected pixel measurement precision for the 3D reconstruction is at the order of one pixel, the system will be considered stable based on all three methods.

**Figure 15.**
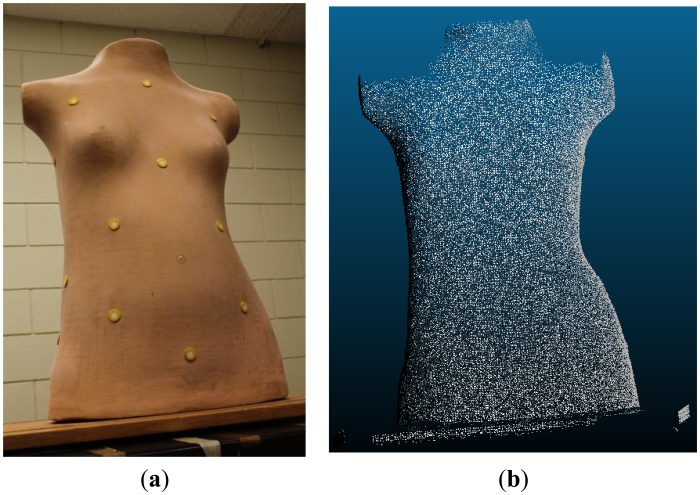
Image of the object of interest (**a**); an example of the reconstructed torso mannequin (**b**).

If the system is considered stable, the reconstruction results will be compatible regardless of which IOP/mounting parameter sets are used. To confirm this hypothesis, and thus to double check the validity of the system stability analysis results, a real image dataset is tested next in order to evaluate the impact on the reconstructed object space. This particular photogrammetric system was designed and built for the 3D reconstruction and evaluation of scoliotic torsos. Thus, a dataset of a scoliotic torso mannequin (see Figure 15a) was used to perform the 3D model reconstruction (see Figure 15b) three times, *i.e.*, once for each set of parameters from the three calibration sessions. Note that the 3D reconstruction was based on sparse image matching at the pixel level.

Each of the resultant 3D models for the torso mannequin was represented either as a point cloud or as a triangular irregular network (TIN). The repeatability between the three models was assessed by registering the point cloud of one model to the TIN of another, where the normal distance between corresponding point-to-patch candidates was minimized via a least-squares adjustment [41]. The average point-to-patch normal distance was 0.52 mm for reconstructed model I *vs*. II, 0.50 mm for reconstructed model I *vs*. III, and 0.54 mm for reconstructed model II *vs*. III. Given the average scale, base-to-depth ratio, and image coordinate measurement precision, the expected plannimetric, *δ_XY_*, and depth, *δ_Z_*, object space precisions were 0.17 mm and 0.41 mm, respectively. Thus the resultant average normal distances were deemed acceptable as the expected total object space precision, *δ_XYZ_*, was 0.48 mm. This confirms the hypothesis that the system is stable, and that the different system calibration parameter sets will not produce significantly different object space.

## 5. Conclusions and Recommendations for Future Work

This paper presented a comprehensive review of the concept of stability analysis for a single camera. It then discussed the calibration and stability analysis for a photogrammetric system comprised of multiple cameras. Three simulation-based methods for performing system stability analysis, which explored the impact of changes in the system calibration parameters on the 3D reconstruction process, were proposed. The first method aimed at reporting image coordinate displacement in the second image of a stereo pair after a combination of forward and backward projections. The second method aimed at identifying the object space parallax, and converting it to image units. The third method directly studied the impact on the normalized image coordinates. A photogrammetric system comprised of multiple cameras was calibrated three times, and the results from the three calibration sessions were used to test the proposed methods for system stability analysis. Since the first method does not consider the changes in IOPs for both cameras simultaneously, and the third method does not consider the changes in the extent of the baseline, the second method is thought of to be the best one, and thus it is the one ultimately recommended for use in system stability analysis. A hypothesis was then drawn that if the system is considered stable, then the changes in the IOPs and mounting parameters coming from different calibration sessions will not affect the 3D reconstruction outcome. An object, for which this photogrammetric system was originally designed and built, was thus reconstructed using the three temporally established sets of IOP and mounting parameters. The three reconstructed 3D models were compared to each other, and confirmed the hypothesis derived from the proposed simulation-based methods for system stability analysis. Thus the contributions of this research work can be summarized as follows:

1. This is the first study that theoretically analyse the stability of a multi-camera system calibration by considering both the IOPs of the individual cameras as well as the mounting parameters relating the camera stations.
2. Rather than relying on a real data for evaluating the stability of the system calibration parameters, the proposed approaches/methodologies are simulation-based. In other words, the proposed methodologies are capable of deriving quantitative measures of the system stability using only the derived system calibration parameters from two epochs.
3. The proposed methodologies not only provide a decision regarding the stability of the system, they also provide a quantitative measure of the impact of changes in the system calibration parameters on the precision as well as the shape of reconstructed objects (*i.e.*, the x and y parallax values reported by the second and third approaches).
4. The proposed methodologies, especially the second one, comprehensively consider the cumulative impact of changes in the IOPs and mounting parameters on the reconstruction process. The only exceptions are the first method, which does not consider the impact of variations in the IOPs of the first camera of the stereo-pair under question, and the third method, that does not consider changes in the magnitude/length of the base line between the camera stations.
5. The proposed methodologies do not require access to the variance covariance matrix of the system calibration parameters or make any assumptions regarding the probabilistic distributions of the available system calibration parameters. Such a characteristic makes the proposed methodologies more practical.
6. The proposed methodologies come with a straight forward procedure for the estimation of the system calibration parameters. The complexity of the proposed system calibration procedure is not affected by the number of utilized cameras and number of involved epochs.
7. The performance of the proposed methodologies and the validity of the underlying hypotheses have been established through experimental results with real data.

Future work will include more testing of the proposed methods: testing on different photogrammetric systems, testing the system stability under different handling conditions, testing the choice of system calibration parameters for the same calibration session, and testing the manufacturing consistency for a particular camera model. Also, as mentioned earlier, if the 3D reconstruction procedure relies on pairwise image matching and tracking of conjugate features, the proposed system stability analysis should be run on every consecutive camera pair. In case of exhaustive image matching, the system stability analysis must be run on every overlapping camera pair. Future work will address stability analysis for 3D reconstruction systems based on triplet matching, simultaneous matching of multiple images (*i.e.*, simultaneous multi-camera stability analysis), or regardless of how the matching is done.

## Figures and Tables

**Table 1.** Interior orientation parameter (IOP) results from the multi-camera system calibration.

	***x_p_* [mm ± mm]**	***y_p_* [mm ± mm]**	***c* [mm ± mm]**	***k*_1_ [mm^−2^ ± mm^−2^]**	***k*_2_ [mm^−4^ ± mm^−4^]**
Camera 1					

Calibration	−0.3241	−0.2208	29.9332	−9.071E-05	2.191E-07
session I	±0.0019	±0.0029	±0.0036	±1.213E-06	±7.250E-09
Calibration	−0.3260	−0.2199	29.9307	−9.148E-05	2.221E-07
session II	±0.0019	±0.0031	±0.0035	±1.339E-06	±8.394E-09
Calibration	−0.3250	−0.2194	29.9288	−8.986E-05	2.157E-07
session III	±0.0020	±0.0031	±0.0035	±1.248E-06	±7.540E-09

Camera 2					

Calibration	0.0030	−0.3266	29.9731	−9.164E-05	2.184E-07
session I	±0.0017	±0.0025	±0.0037	±1.223E-06	±7.701E-09
Calibration	0.0028	−0.3260	29.9733	−9.118E-05	2.127E-07
session II	±0.0017	±0.0024	±0.0034	±1.150E-06	±6.983E-09
Calibration	0.0020	−0.3265	29.9754	−9.072E-05	2.153E-07
session III	±0.0018	±0.0026	±0.0039	±1.265E-06	±8.000E-09

Camera 3					

Calibration	−0.0600	−0.1356	30.1074	−8.893E-05	2.016E-07
session I	±0.0017	±0.0023	±0.0030	±1.155E-06	±7.428E-09
Calibration	−0.0639	−0.1375	30.1062	−8.954E-05	2.045E-07
session II	±0.0017	±0.0024	±0.0031	±1.115E-06	±7.159E-09
Calibration	−0.0653	−0.1364	30.1113	−8.861E-05	2.036E-07
session III	±0.0017	±0.0023	±0.0031	±1.130E-06	±7.168E-09

Camera 4 (reference)					

Calibration	−0.0711	−0.3004	29.6863	−9.512E-05	2.186E-07
session I	±0.0016	±0.0020	±0.0027	±1.091E-06	±7.081E-09
Calibration	−0.0716	−0.3029	29.6892	−9.540E-05	2.162E-07
session II	±0.0016	±0.0020	±0.0026	±1.054E-06	±6.683E-09
Calibration	−0.0746	−0.3007	29.6862	−9.476E-05	2.195E-07
session III	±0.0016	±0.0020	±0.0027	±1.120E-06	±7.302E-09

Camera 5					

Calibration	−0.1933	−0.2791	29.6769	−1.003E-04	2.249E-07
session I	±0.0015	±0.0020	±0.0026	±1.060E-06	±6.781E-09
Calibration	−0.1965	−0.2843	29.6787	−1.000E-04	2.198E-07
session II	±0.0015	±0.0021	±0.0028	±1.097E-06	±7.053E-09
Calibration	−0.1969	−0.2759	29.6779	−9.740E-05	2.111E-07
session III	±0.0015	±0.0020	±0.0028	±1.092E-06	±6.910E-09

Camera 6					

Calibration	0.0058	−0.1936	29.9178	−9.479E-05	2.291E-07
session I	±0.0015	±0.0019	±0.0026	±9.549E-07	±5.710E-09
Calibration	0.0043	−0.1950	29.9198	−9.530E-05	2.302E-07
session II	±0.0016	±0.0020	±0.0030	±9.634E-07	±5.737E-09
Calibration	0.0029	−0.1944	29.9169	−9.434E-05	2.286E-07
session III	±0.0016	±0.0020	±0.0028	±9.988E-07	±5.932E-09

Camera 7					

Calibration	−0.0006	−0.2094	30.3515	−8.691E-05	2.008E-07
session I	±0.0016	±0.0022	±0.0029	±1.076E-06	±6.540E-09
Calibration	−0.0037	−0.2114	30.3532	−8.714E-05	1.995E-07
session II	±0.0016	±0.0023	±0.0031	±1.064E-06	±6.414E-09
Calibration	−0.0029	−0.2068	30.3562	−8.654E-05	2.024E-07
session III	±0.0017	±0.0023	±0.0032	±1.139E-06	±6.939E-09

**Table 2.** Mounting parameter results from the multi-camera system calibration.

	***b_X_* [m ± mm]**	***b_Y_* [m ± mm]**	***b_Z_* [m ± mm]**	***b_ω_* [° ± ″]**	***b_φ_* [° ± ″]**	***b _κ_* [° ± ″]**
Camera 1						

Calibration	−0.0081	0.7654	−0.4714	−44.6643	0.1286	−0.0186
session I	±0.04	±0.29	±0.23	±22.59	±14.08	±8.04
Calibration	−0.0081	0.7653	−0.4715	−44.6625	0.1319	−0.0167
session II	±0.04	±0.28	±0.22	±23.28	±14.17	±7.91
Calibration	−0.0081	0.7653	−0.4715	−44.6612	0.1238	−0.0223
session III	±0.04	±0.29	±0.23	±23.70	±14.72	±8.05

Camera 2						

Calibration	−0.0104	0.5651	−0.2129	−28.6312	0.3731	−3.5568
session I	±0.04	±0.21	±0.18	±20.30	±14.12	±5.80
Calibration	−0.0104	0.5651	−0.2130	−28.6299	0.3735	−3.5554
session II	±0.03	±0.21	±0.17	±19.78	±13.80	±5.69
Calibration	−0.0105	0.5652	−0.2128	−28.6314	0.3675	−3.5592
session III	±0.04	±0.22	±0.19	±21.08	±14.68	±5.83

Camera 3						

Calibration	−0.0121	0.3038	−0.0605	−15.9938	−0.1045	1.4400
session I	±0.03	±0.12	±0.15	±19.56	±14.18	±3.91
Calibration	−0.0121	0.3037	−0.0606	−15.9921	−0.0991	1.4409
session II	±0.03	±0.12	±0.15	±19.84	±14.07	±3.84
Calibration	−0.0121	0.3038	−0.0603	−15.9927	−0.1031	1.4384
session III	±0.03	±0.12	±0.16	±19.92	±14.34	±3.92

Camera 4 (reference)						

Calibration	0.0000	0.0000	0.0000	0.0000	0.0000	0.0000
session I	N/A	N/A	N/A	N/A	N/A	N/A
Calibration	0.0000	0.0000	0.0000	0.0000	0.0000	0.0000
session II	N/A	N/A	N/A	N/A	N/A	N/A
Calibration	0.0000	0.0000	0.0000	0.0000	0.0000	0.0000
session III	N/A	N/A	N/A	N/A	N/A	N/A

Camera 5						

Calibration	0.0039	−0.2946	−0.0621	13.4939	1.7349	5.1812
session I	±0.03	±0.11	±0.14	±18.39	±13.94	±3.35
Calibration	0.0039	−0.2946	−0.0622	13.4904	1.7417	5.1822
session II	±0.03	±0.11	±0.14	±18.42	±13.87	±3.30
Calibration	0.0039	−0.2946	−0.0620	13.4978	1.7349	5.1820
session III	±0.03	±0.11	±0.15	±18.54	±14.17	±3.39

Camera 6						

Calibration	0.0303	−0.5536	−0.2125	28.2217	1.4085	1.6045
session I	±0.03	±0.21	±0.16	±18.08	±12.93	±5.44
Calibration	0.0304	−0.5535	−0.2126	28.2228	1.4132	1.6043
session II	±0.04	±0.21	±0.16	±18.05	±13.07	±5.38
Calibration	0.0303	−0.5536	−0.2125	28.2204	1.4101	1.6070
session III	±0.03	±0.21	±0.16	±18.62	±13.39	±5.49

Camera 7						

Calibration	0.0441	−0.7414	−0.4620	42.6999	3.8138	3.4004
session I	±0.04	±0.28	±0.22	±19.05	±12.62	±7.55
Calibration	0.0441	−0.7413	−0.4621	42.6995	3.8196	3.4001
session II	±0.04	±0.28	±0.22	±18.79	±12.40	±7.43
Calibration	0.0441	−0.7415	−0.4618	42.7011	3.8138	3.4042
session III	±0.04	±0.28	±0.22	±19.64	±13.10	±7.59

**Table 3.** Results from the system stability analysis.

	**Method 1**			**Method 2**			**Method 3**		

	**RMSE_x_ [px] (┴ baseline)**		**RMSE_y_ [px] (∥ baseline)**	**RMSE_x_ [px] (┴ baseline)**		**RMSE_y_ [px] (∥ baseline)**	**RMSE_x_ [px] (┴ baseline)**		**RMSE_y_ [px] (∥ baseline)**
Calibration session I *vs.* calibration session II									

Cameras 1 & 2	0.34		0.21	0.03		0.21	0.18		0.56
Total RMSE [px]		0.40			0.21			0.59	
Cameras 2 & 3	0.11		0.18	0.10		0.07	0.30		0.43
Total RMSE [px]		0.21			0.12			0.53	
Cameras 3 & 4	0.66		0.60	0.07		0.20	0.03		0.10
Total RMSE [px]		0.89			0.21			0.10	
Cameras 4 & 5	0.14		0.44	0.05		0.15	0.07		0.23
Total RMSE [px]		0.46			0.16			0.24	
Cameras 5 & 6	0.55		1.05	0.06		0.13	0.13		0.26
Total RMSE [px]		1.18			0.15			0.29	
Cameras 6 & 7	0.39		0.29	0.05		0.17	0.06		0.04
Total RMSE [px]		0.48			0.18			0.08	
Cameras 1 & 2	0.21		0.23	0.04		0.22	0.30		0.80
Total RMSE [px]		0.31			0.23			0.85	
Cameras 2 & 3	0.42		0.44	0.30		0.27	0.20		0.42
Total RMSE [px]		0.61			0.41			0.47	
Cameras 3 & 4	0.85		0.12	0.17		0.04	0.26		0.35
Total RMSE [px]		0.86			0.17			0.44	
Cameras 4 & 5	0.62		0.06	0.08		0.05	0.43		0.38
Total RMSE [px]		0.62			0.10			0.57	
Cameras 5 & 6	0.58		0.99	0.20		0.41	0.37		0.53
Total RMSE [px]		1.15			0.45			0.65	
Cameras 6 & 7	0.83		0.05	0.23		0.25	0.06		1.24
Total RMSE [px]		0.83			0.34			1.24	

Calibration session II *vs.* calibration session III									

Cameras 1 & 2	0.15		0.37	0.02		0.42	0.12		0.24
Total RMSE [px]		0.40			0.42			0.27	
Cameras 2 & 3	0.31		0.27	0.20		0.23	0.10		0.10
Total RMSE [px]		0.41			0.31			0.14	
Cameras 3 & 4	0.28		0.65	0.11		0.22	0.27		0.39
Total RMSE [px]		0.71			0.24			0.47	
Cameras 4 & 5	0.53		0.49	0.06		0.20	0.48		0.60
Total RMSE [px]		0.72			0.21			0.77	
Cameras 5 & 6	0.05		2.04	0.14		0.54	0.25		0.78
Total RMSE [px]		2.04			0.56			0.82	
Cameras 6 & 7	0.45		0.28	0.19		0.42	0.08		1.21
Total RMSE [px]		0.53			0.46			1.21	
